# Regulation of the Function and Expression of EpCAM

**DOI:** 10.3390/biomedicines12051129

**Published:** 2024-05-20

**Authors:** Di Xiao, Mingrui Xiong, Xin Wang, Mengqing Lyu, Hanxiang Sun, Yeting Cui, Chen Chen, Ziyu Jiang, Fan Sun

**Affiliations:** 1 College of Life Sciences and Health, Wuhan University of Science and Technology, Wuhan 430081, China; 1dxiao@wust.edu.cn (D.X.); mingruixiong97@wust.edu.cn (M.X.); xinwang@wust.edu.cn (X.W.); mengqinglyu@wust.edu.cn (M.L.); hanxiangsun2023@wust.edu.cn (H.S.); yetingcui09@wust.edu.cn (Y.C.); 2 Institute of Biology and Medicine, Wuhan University of Science and Technology, Wuhan 430081, China; 3 Tumor Precision Diagnosis and Treatment Technology and Translational Medicine, Hubei Engineering Research Center, Zhongnan Hospital of Wuhan University, Wuhan 430071, China; chenchenvskt@whu.edu.cn

**Keywords:** EpCAM, structure, function, expression, therapy

## Abstract

The epithelial cell adhesion molecule (EpCAM) is a single transmembrane protein on the cell surface. Given its strong expression on epithelial cells and epithelial cell-derived tumors, EpCAM has been identified as a biomarker for circulating tumor cells (CTCs) and exosomes and a target for cancer therapy. As a cell adhesion molecule, EpCAM has a crystal structure that indicates that it forms a cis-dimer first and then probably a trans-tetramer to mediate intercellular adhesion. Through regulated intramembrane proteolysis (RIP), EpCAM and its proteolytic fragments are also able to regulate multiple signaling pathways, Wnt signaling in particular. Although great progress has been made, increasingly more findings have revealed the context-specific expression and function patterns of EpCAM and their regulation processes, which necessitates further studies to determine the structure, function, and expression of EpCAM under both physiological and pathological conditions, broadening its application in basic and translational cancer research.

## 1. Introduction

In 1979, Herlyn and colleagues discovered a humoral antigen recognized by the monoclonal antibody 1083-17-1A and named it (CO)17-1A [1]. Subsequently, this antigen was assigned different nomenclatures, including TROP-1 [2], EGP40 [3], CD326 [4], and the latest and widely used EpCAM [5]. Initially, EpCAM was found ubiquitously and strongly expressed on the surface of various epithelial cancer cells, in particular in prostate, pancreatic, and colorectal cancers [6]. Given its high expression level and immunogenicity, EpCAM is considered as a surface biomarker and potential therapeutic target in human cancers [7].

This review provides a brief overview of the structure and function of EpCAM, highlighting its controversial roles under both physiological and pathological conditions. Next, signaling pathways involving EpCAM, in particular the classical nuclear signaling pathway, are summarized, which improves the understanding of EpCAM’s function and application in the field of cell biology. Finally, the dynamic expression pattern of EpCAM and its regulation are discussed in a cell- and a tissue-specific manner.

## 2. Structure and Function of EpCAM

### 2.1. Structure of the EpCAM Monomer

**Figure 1:**
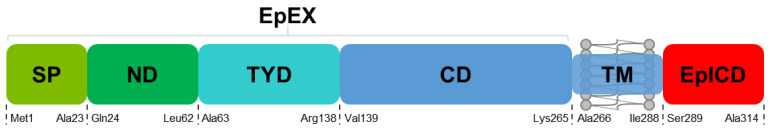
Schematic of the EpCAM protein. EpEX, extracellular domain; SP, signal peptide; ND, N-terminal domain; TYD, thyroglobulin type 1A domain; CD, C-terminal domain; TM, transmembrane domain; EpICD, intracellular domain.

Human EpCAM is a single transmembrane protein of 314 amino acids (aa) [8], comprising an extracellular domain (EpEX) of 265 aa, a transmembrane domain (TM) of 23 aa, and an intracellular domain (EpICD) of 26 aa. As a transmembrane protein, the maturation of EpCAM requires the removal of the signal peptide at Ala23 and in rare cases at Ala21 [9,10]. After shearing off the signal peptide, EpEX can be subdivided into three regions: the cysteine-rich N-terminal domain (ND), the thyroglobulin type 1A domain (TYD), and the cysteine-free C-terminal domain (CD) (Figure 1). Initially, both ND and TYD were regarded as two tandem epidermal growth factor (EGF)-like domains. However, subsequent analysis on the disulfide bond and glycosylation indicated that ND has a unique disulfide bond pattern that is different from the EGF-like domain, whereas TYD has a disulfide bond pattern similar to methyglobulin type 1A (TY1A) [10]. These differences were confirmed by the crystal structure of the extracellular domain of EpCAM, which further suggested that the three domains of EpEX are in contact with each other to form a triangular shape [11]. In addition, the extracellular domains of the EpCAM dimer are in a heart-shaped form [11].

After the cleavage of the signal peptide, the ND region of EpCAM forms three distinct disulfide bonds (Cys27-Cys46, Cys29-Cys59, Cys38-Cys48), which are far from the plasma membrane, making them an ideal target site for EpCAM antibodies [10,12,13]. The similarity of the disulfide bond patterns between TYD and TY1A probably allows EpCAM to perform as an inhibitor of cathepsin L (CTSL) [10,14]. However, the CTSL inhibitor function of TYD in normal cells needs to be clarified because the EpCAM dimer, the major form, is unfavorable for substrate binding, due to steric hindrance [11], and the EpCAM monomer, the minor form, degrades rapidly [15]. In addition, matriptase is able to mediate the cleavage between Arg80 and Arg81 in the TYD region [16]. Finally, the CD region of EpCAM does not harbor any Cys residue and disulfide bond. The function of the CD region remains unclear, and it may provide flexibility to extracellular structures [17]. Moreover, the CD region can be cleaved through regulated intramembrane proteolysis (RIP) at the α-site (D243/P244, P244/G245) by a disintegrin and metalloproteinase 17 (ADAM17), also known as tumor necrosis factor-α-converting enzyme (TACE), and at the β-site (Y250/Y251, Y251/Y252) by β-secretase 1 (BACE1) [18].

Initially, only N-glycosylation was found in human EpEX whereas no O-glycosylation was observed [8]. There are three specific sites subject to N-glycosylation: Asn74, Asn111, and Asn198 [10]. It is worth noting that the degree of N-glycosylation at these sites varies among species. For example, in insect cells, partial N-glycosylation occurs at Asn74, whereas there is complete N-glycosylation at Asn111 and no N-glycosylation at Asn198 [10]. In contrast, in mammalian cells, Munz et al. demonstrated that all three glycosylation sites undergo N-glycosylation, and N-glycosylation at Asn198 is critical for the stability and membrane expression of EpCAM [19]. The mutation of Asn198 to Ala causes a remarkable reduction in the cell surface EpCAM (about 55%) and decreases the half-life from 21 to 7 h. Due to an improvement in detection technology, O-glycosylation was also found at Thr171 and Thr172 within EpCAM recently, but the function of this modification has not yet been determined [20].

The TM region of EpCAM is rich in valine but not leucine that is frequently observed in other transmembrane domains [21]. In line with this structure, multiple RIP cleavages have been demonstrated at valine in the TM region of EpCAM, including three γ-sites (V273/V274, V274/V275, V275/V276) and two ε-sites (V284/V285, L286/V287) [18,22]. The intact TM region plays a critical role in maintaining the structure and function of EpCAM. On the one hand, the TM region is required for the dimerization of EpCAM by its α-helix structure, which subsequently prevents EpCAM from cleavage and degradation [11]. On the other hand, the TM region accounts for the protein–protein interaction between EpCAM and claudin-7. Both deletion of the TM region and mutation of the AxxxG motif within the TM region result in the loss of interaction between EpCAM and claudin-7. However, it is noteworthy that the binding of the TM region to claudin-7 also inhibits the oligomerization of EpCAM [23]. In addition, the TM region has been found to be associated with tetraspanin-enriched domains (TEMs) [24], which is dependent on its interaction with claudin-7 [23,25].

As a cell adhesion molecule, EpCAM interacts with the cytoskeleton via EpICD. Balzar et al. discovered two α-actinin-binding sites within EpICD (289~296 aa, 304~314 aa) and revealed that the direct interaction between EpICD and α-actinin has an impact on the subcellular localization of EpCAM [26]. Furthermore, Schnell et al. proposed that EpICD contains a putative PDZ domain-binding motif (312-LNA-314: Leu312, Asn313, and Ala314) that is often found in other PDZ domain-interacted proteins [27]. However, the direct interaction of EpICD with PDZ domain-containing protein(s) needs to be addressed to support the hypothesis [28]. Finally, it was noted that the free EpICD fragment produced by RIP hydrolysis works together with other factors to control gene expression and promote cell proliferation [29].

### 2.2. Oligomeric Model of EpCAM

EpCAM was first described as a homophilic calcium-independent cell adhesion molecule; however, the molecular structure of EpCAM, which mediates adhesion among cells, has remained elusive [3,5]. Scientists later proposed two models. One hypothesis suggests that EpCAM forms a cis-homodimer on the plasma membrane of one celland then forms a trans-tetramer with neighboring cells to mediate intercellular adhesion [30]. The other one states that EpCAM cis-tetramers are able to trans-interact with each other to form trans-octamers [31]. Recently, the first model was proved by the crystal structure analysis of EpCAM, indicating the requirement of EpCAM cis-dimerization for trans-tetramer formation in cell–cell adhesion [11]. However, the trans-tetramer of EpCAM is hard to detect by current techniques [11,32]. The lack of direct evidence of an EpCAM trans-tetramer questions the role of EpCAM in cell adhesion (see Section 2.3). More information about the two adhesion models for EpCAM is available in the review by Gaber et al. [33]. In conclusion, although the trans-tetramer remains a theoretical structure, EpCAM is confirmed predominantly as a cis-dimer on the cell surface. There is an urgent need for more investigation to determine the structure of EpCAM oligomers in the future.

### 2.3. EpCAM Mediates Cell Adhesion

There is no doubt that EpCAM is involved in intercellular adhesion, yet its mechanism remains in debate. The general idea suggests that EpCAM functions as a non-classical adhesion molecule to regulate intercellular adhesion junctions by interacting with classical adhesion molecules, e.g., cadherin. Litvinov et al. demonstrated that the overexpression of EpCAM weakens E-cadherin-mediated cell adhesion without affecting the total amount of E-cadherin [34]. These data suggest the competitive binding of the two adhesion systems to α-catenin. However, this explanation lacks strong evidence, as EpCAM does not directly bind to α-catenin [31]. Although no molecule has been characterized to support this hypothesis, it has been proved that these two adhesion systems compete with each other during cytoskeletal remodeling. The direct binding of EpCAM’s EpICD to α-actinin [26], as well as the interruption of the interaction between α-catenin and F-actin by EpCAM [35], suggests that EpCAM inhibits cadherin-mediated adhesion during cytoskeletal remodeling. Winter et al. demonstrated that EpCAM interacts with the PI3K regulatory subunit p85, resulting in a transfer of PI3K from N-cadherin to EpCAM, leading to the dissociation of the cadherin adhesion complex in epithelial cells [36]. It is important to note that competition between the two types of adhesion exists for a favorable outcome of functional adhesion, rather than mere antagonism. According to the findings of Guerra et al., dysfunction of both E-cadherin and β-catenin is observed in the intestines of *Epcam*-deficient mice, resulting in the partial impairment of adhesion junctions [37]. The collaboration and competition between EpCAM and cadherin suggest a coordinated balance between different adhesion systems under physiological conditions.

EpCAM facilitates the formation and repair of tight junctions (TJs) by recruiting claudin-7. The direct interaction between EpCAM and claudin-7 was first discovered by Ladwein et al. [38]. Later, Lei et al. reported that in the absence of EpCAM, the recruitment of claudin-7 to the tight junction is greatly diminished, leading to the loss of the TJ [39]. These findings indicate that interaction between EpCAM and claudin-7 is required for the recruitment of claudin-7 to the TJ and junction formation. In contrast, a recent study by Higashi et al. revealed that once TJ disruption occurs, the EpCAM–claudin-7 complex in the basolateral membrane moves to the apical membrane and is hydrolyzed by membrane-anchored serine proteinases (MASPs) to release claudin-7, which, in turn, repairs the damage [40]. However, it is worth noting that EpCAM-deficient cells do not experience impaired TJ formation in the epithelial barrier, indicating alternative mechanisms involved in TJ formation [40]. Hence, EpCAM mainly contributes to the repair of pre-existing TJs. In line with this hypothesis, MASP, albeit in small amounts, is effective in cleaving EpCAM [16], which is also consistent with the concept that EpCAM partially contributes to TJ repair. In summary, these studies suggest that EpCAM is involved in the formation and repair of tight junctions. However, the underlying mechanism of the movement of EpCAM and its complex from the basolateral membrane to the apical membrane remains to be addressed.

However, recent findings strongly challenge EpCAM as a non-classical cell adhesion molecule for mediating cell adhesion. There are two main points to be considered. First, neither the depletion of EpCAM nor the fragmentation of EpCAM in a wide range of cell lines is able to affect their adhesion to the extracellular matrix and neighboring cells [22,41]. Second, only the EpCAM cis-dimer but not the trans-tetramer has been detected by various existing models and methods [32]. Although the latter can be weakly explained by intercellular heterogeneity and inadequate detection techniques, Gaber et al. and Fagotto et al. conclude that EpCAM is unlikely to serve as a cell adhesion molecule in view of its crystal structure and the definition of CAM [28,33].

Though the function of EpCAM as a non-classical cell adhesion molecule has been challenged, there is no doubt about its role in regulating cell adhesion. Fagotto et al. proposed that EpCAM functions as a heterophilic cell adhesion molecule or indirectly regulates cell adhesion through inhibiting the PKC/ERK signaling pathway independently of its CAM activity [28]. The former conjecture suggests that there is an unknown cell adhesion molecule ubiquitously expressed in epithelial cells to interact with EpCAM. The latter seems more convincing due to increasing evidence indicating EpCAM regulation of multiple signal transduction.

## 3. Signal Transduction of EpCAM

### 3.1. The Nuclear Signaling Pathway of EpCAM Is Mediated by RIP

**Figure 2:**
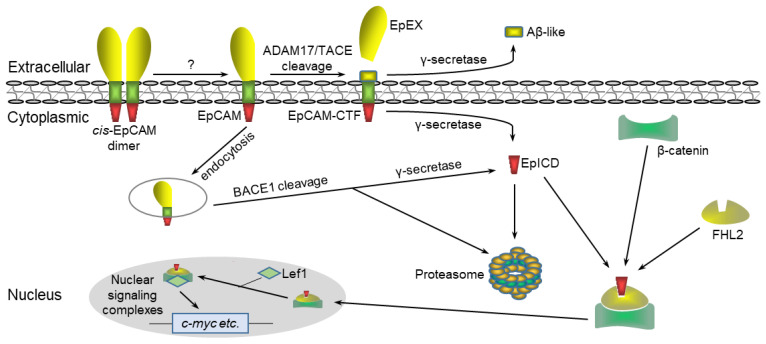
Schematic of the nuclear signaling pathway of RIP-mediated EpCAM. Initially, it is still unclear how the EpCAM dimer dissociates into monomers, which undergoes two-step enzymatic cleavage by RIP, resulting in a functional intracellular EpICD fragment. This fragment subsequently complexes with FHL2 and β-catenin and enters the nucleus to form a nuclear signaling complex with transcription factor LEF1. Finally, the nuclear signaling complex binds to the promoter of target genes to initiate transcription.

In 2009, Maetzel et al. systematically proposed extracellular–nuclear signaling transduction for EpCAM, demonstrating that EpCAM is cleaved by RIP to release an EpICD fragment, which, in turn, interacts with components of the Wnt pathway to initiate gene expression in cancer cells (Figure 2) [29]. Here is a brief overview of the nuclear signaling of EpCAM, while more information is provided in the work of Gires et al. [42].

There are three major steps in the cleavage of EpCAM by RIP. First, EpCAM is cleaved by ADAM17/TACE at the α-site or by BACE1 at the β-site, respectively, to produce soluble EpEX and EpCTF, a C-terminal fragment of EpCAM, that remains in the plasma membrane [18,29]. Second, the membrane EpCTF is hydrolyzed at both γ- and ε-sites by γ-secretase, which contains presenilin-2 as the catalytic subunit. The cleavage at the γ-site generates a soluble Aβ-like fragment, whose biological function needs further investigation [43]. Meanwhile, the cleavage at the ε-site leads to the release of the EpICD fragment into the cytoplasm. Finally, the cytoplasmic EpICD fragment complexes with FHL2 and β-catenin and then translocates into the nucleus to bind with the transcription factor LEF1, which collaboratively activates the transcription of the genes involved in cell proliferation, e.g., *c-MYC*, *CCNA2*, *CCND1*, and *CCNE1* (Figure 2) [29,44,45].

Despite the comprehensive elucidation of the nuclear signaling pathway of EpCAM, several issues remain. The first is the dissociation of the EpCAM dimer. While EpCAM typically exists as a dimer, its TM domain tends to form a cis-dimer (Figure 2), which is not easy for ADAM17/TACE to access and cleave [11]. During the in vitro purification of EpCAM, more EpCAM monomers are retrieved under acidic conditions, which can be reversed by increasing the pH, indicating that an acidic microenvironment may be necessary for the dissociation of the EpCAM dimer [46]. Unfortunately, there is no study yet addressing the question of how the EpCAM dimer dissociates into monomers during nuclear signaling. Second, the low efficiency of enzyme cleavage should be taken into account. Membrane EpCAM is cleaved by ADAM17/TACE in the extracellular space; however, high expression of ADAM17/TACE is observed only in some cancer cells [29]. In contrast, BACE1 is widely expressed and is predominantly localized in the trans-Golgi network. However, BACE1 is only active in the acidic environment of endosomes and lysosomes, where the cleavage of EpCAM occurs after endocytosis [18]. In the context of the acidic microenvironment of cancer, BACE1 may cleave EpCAM at the β-site extracellularly [22]. Additionally, γ-secretase is released at a slow rate. Moreover, only 50% of EpCTF, whether exogenous or endogenous, is cleaved by γ-secretase within 4.75 h [15]. Meanwhile, it is worth noting that most EpICD fragments (94%~99%) are subjected to proteasomal degradation but do not initiate nuclear signaling [15,18]. Accordingly, two ubiquitination sites, Lys299 and Lys303, have been identified within the EpICD region [47]. The third is how RIP is activated and terminated properly. Initially, Maetzel et al. proposed that the homophilic interaction of EpCAM between neighboring cells activates RIP to generate soluble EpEX, which is able to bind to EpCAM to further activate RIP [29]. However, the ability of EpCAM to mediate trans-homophilic adhesion remains unclear. Recently, several studies have demonstrated that EpEX can function as a ligand for EGFR to activate the EGF/EGFR/ERK pathway, which, in turn, induces RIP [48], evidenced by the phosphorylation of ADAM17/TACE and γ-secretase [49]. However, the origin of free EpEX and how RIP terminates the EpCAM cleavage remain unclear. One recent study indicated that double-negative feedback regulation exists between EpCAM and ERK [50]. EpCAM overexpression has been found to inhibit ERK activity in various cancer cell lines, while ERK is also capable of directly and indirectly suppressing EpCAM transcription. Further studies will focus on the mechanism of EpCAM inhibition of ERK activity and the distinct roles of EpCAM and EpEX in the regulation of ERK activity. In conclusion, all these studies indicate that the enzymatic efficiency of RIP is suboptimal, which results in only a small amount of EpICD being involved in nuclear signaling. It also suggests that EpCAM nuclear signaling is fine-tuned by RIP in the long term.

### 3.2. Other Signaling Pathways

EpCAM not only plays a crucial role in the Wnt signaling pathway through the EpICD fragment but also modulates multiple signaling pathways by other proteolytic fragments and even intact EpCAM. Notably, zebrafish EpEX functions as a core structure to derepress Wnt and collaboratively activate Wnt2bb signaling in endodermal cells [51]. Furthermore, EpEX, as a ligand of EGFR, is capable of activating the EGF/EGFR signaling pathway in head and neck cancer cells [48]. A collaboration of EGF and EpEX in epithelial–mesenchymal transition (EMT) induction is observed in endometrial cancer cells [52]; however, the opposite result occurs in head and neck cancer cells [48]. This phenomenon is believed to be involved in the different degrees of ERK1/2 phosphorylation induced by EpEX and EGF [48]. In comparison to EGF, EpEX induces weaker ERK1/2 phosphorylation downstream of the EGF/EGFR pathway. Thus, EpEX is able to competitively bind to EGFR, impeding the activation of EMT-associated transcription factors by EGF.

Recently, Fagotto et al. hypothesized that EpCAM regulates cell adhesion and migration through the EpCAM/nPKC/myosin pathway [28,53]. The requirement of EpCAM for cell adhesion and migration has also been demonstrated in embryonic development in zebrafish and African clawed frog, indicating a mechanism independent of CAM [54,55]. Further gain-of-function/loss-of-function experiments have characterized that a sequence close to the membrane in EpICD can serve as a pseudo-substrate for nPKC, efficiently blocking its activity and modulating myosin contractility [56]. It is also observed that only intact EpCAM, not its truncated forms, is capable of repairing the damaged epithelium [56]. Additionally, previous research has revealed that EpCAM forms a complex with p85, the regulatory subunit of PI3K [36], and promotes tumor progression in prostate cancer and nasopharyngeal carcinoma through the regulation of the PI3K/AKT/mTOR pathway [57,58,59]. Recently, Yang et al. discovered reduced phosphorylation levels of PI3K, AKT, and mTOR in breast cancer cells with deficiency in EpCAM N-glycosylation [60], which was confirmed by Wen et al. [61]. In summary, these data suggest that after N-glycosylation, EpCAM binds to p85 to activate the PI3K/AKT/mTOR pathway, and the role of other post-translational modifications (PTMs), e.g., O-glycosylation, needs to be explored in the future.

## 4. Expression of EpCAM and Its Regulation

### 4.1. Expression of EpCAM in Normal and Cancerous Tissues

In the early stage of development, EpCAM is ubiquitously expressed and has been extensively investigated in several animal models, such as African clawed frog, zebrafish, and mice. Both mRNA and protein levels of EpCAM are detected in all germ layers during the early development of the African clawed frog, with the highest level in the ectoderm, whereas depletion of EpCAM significantly impairs cell motility, leading to deficiency in embryonic development [55,56]. In contrast, although only mRNA was detected, a recent study on zebrafish showed that EpCAM is ubiquitously expressed in pre-gastrulation embryos but only detected on the surface of the ectoderm in gastrulation embryos [54]. The status of EpCAM expression in other embryonic layers of zebrafish remains elusive. In mouse blastocysts, the expression of EpCAM is observed in both the ectoderm and the endoderm but is repressed in the mesoderm [62]. Additionally, along with embryonic development, EpCAM remains expressed in the epithelial tissues of the ectoderm and the endoderm but is significantly downregulated in the neural ectoderm and the mesoderm and its derived tissues. The spatiotemporal expression pattern of EpCAM contributes to the early separation of endoderm and mesoderm clusters, representing a novel mechanism to regulate the differentiation of embryonic stem cells [62]. Moreover, EpCAM expression is detected in the trophectoderm, giving it the name trophectodermal surface antigen-1 (TROP1) [2], as well as in germ cells throughout all development stages [41].

In well-differentiated tissues, EpCAM is predominantly expressed in the basolateral membrane of the pseudostratified ciliated columnar and transitional epithelium but not the squamous stratified epithelium [63]. The organ/tissue with the strongest EpCAM expression is the colorectum, which develops congenital tufted enteropathy (CTE) due to EpCAM mutation in humans [64]. It is noted that the expression level of EpCAM is dependent on the status of cell differentiation. Generally, highly differentiated cells exhibit less expression of EpCAM, and vice versa. For example, EpCAM is strongly expressed in adult hepatic stem cells and embryonic liver tissues in humans but is rare in highly differentiated hepatocytes [63,65]. The high expression of EpCAM in poorly differentiated cells probably suggests its involvement in promoting or maintaining cell stemness. Accordingly, EpCAM is found to be strongly expressed on the surface of undifferentiated hESCs and serves as a surface marker for both human and mouse embryonic stem cells, likely to facilitate proliferation and differentiation [66,67]. In contrast, the transcriptional levels of *EPCAM* rapidly decrease following the differentiation of hESCs [68], which is accompanied by a decrease in the expression of *c-MYC*, *OCT3/4*, and *STAT3* [69]. Further studies have shown that *EPCAM* can regulate the expression of the key factors of cell reprogramming, e.g., *OCT4*, *SOX2*, and *NANOG*, indicating that the coexpression network architecture of *EPCAM*, *NANOG*, and *SOX2* is necessary to induce the reprogramming of somatic cells to pluripotent stem cells (iPSCs) [70]. In cancer cells, EpCAM has proven to be a stem cell marker and maintains and promotes cell stemness in a range of cancers, including hepatocellular carcinoma [71], nasopharyngeal carcinoma [72], non-small-cell lung cancer [73], colorectal cancer [74], and gastric cancer [75]. In conclusion, the expression of EpCAM is strongly associated with the expression of reprogramming factors, which play a vital role in maintaining cell stemness. This can provide novel and more effective approaches for generating iPSCs and targeted therapy against tumor stem cells in the future.

Generally, EpCAM is ubiquitously expressed in cancer tissues of epithelial origin while showing low levels or even being absent in lymphomas, melanomas, and other tumors derived from mesenchymal or neural tissues [6]. It is highly expressed in breast [76], gastric [77], pancreatic [78], prostate [79,80], and colorectal [81] cancers, with the strongest expression observed in colorectal cancer [6]. In contrast, limited expression of EpCAM is found in renal clear cell carcinoma, hepatocellular carcinoma, and glioma [6,82]. With respect to metastatic cancers, the expression of EpCAM is higher than that in primary tumors, including prostate cancer [80] and breast cancer [83]. This indicates that EpCAM may promote EMT to facilitate tumor metastasis. However, the role of EpCAM in EMT is multifaceted and sometimes conflicting. Detailed information about the function of EpCAM in EMT is reviewed by Brown et al. [84]. Moreover, the expression of EpCAM varies in the subtypes of the same cancer. For example, compared with other subtypes, reduced expression of EpCAM is detected in the lobular subtype of breast cancer [82,85,86]. Despite the consistent phenomenon, a reasonable explanation to determine the regulation of EpCAM expression remains elusive. In summary, the spatiotemporal expression pattern of EpCAM is context specific, as is the regulation mechanism.

### 4.2. Expression of EpCAM in Circulating Tumor Cells (CTCs) and Exosomes

Metastasis is the major cause of death among cancer patients. Metastatic tumor cells in the circulatory system are considered as circulating tumor cells (CTCs), which can be detected and captured through EpCAM, the surface marker ubiquitously and strongly expressed in epithelial cancer cells [6,87,88]. In fact, a CTC-counting kit based on EpCAM-positive labeling has been approved by the FDA [89]. At the beginning of metastasis, epithelial cancer cells often undergo EMT for their aggressiveness [90]. EpCAM-positive CTCs with a mesenchymal phenotype are more invasive than EpCAM-negative mesenchymal CTCs in mouse models of both metastatic breast cancer and prostate cancer [91,92]. Consequently, a higher level of EpCAM-positive CTCs in the blood is often correlated with worse prognosis. Hence, EpCAM-positive CTCs have been used as a prognostic biomarker in patients with various cancer types, including lung cancer [93], hepatocellular carcinoma [94], prostate cancer [95], neuroendocrine cancer [96], breast cancer [97], and colorectal cancer [98]. However, limitations exist in the EpCAM-positive CTC assessment method, due to its insensitiveness to CTCs with low EpCAM expression. In addition, the expression of EpCAM is low or absent in CTCs in certain cancers, e.g., EpCAM-negative CTCs in metastatic breast cancer [99]. As a result, other biomarkers are often evaluated simultaneously, including claudin-4 assay, to circumvent the weakness of EpCAM-positive CTC assessment in malignant mesotheliomas [100].

Exosomes are extracellular bilayer lipid vesicles naturally secreted from cells, containing proteins, nucleic acids, lipids, and metabolites to mediate cell-to-cell communication and regulate the behavior of target cells [101]. Through anti-EpCAM-coupled magnetic beads, exosomes are isolated and enriched in human colorectal cancer cells [102], ovarian cancer cells [103,104], and breast cancer cells [105]. Compared with healthy tissues, higher levels of EpCAM-positive exosomes are detected in the blood of patients with lung cancer [106] and colorectal cancer [107]. Moreover, the content of EpCAM-positive exosomes is positively correlated with cancer cell invasion, conferring on its a staging biomarker in ovarian cancer [108]. However, the mechanism to recruit EpCAM to vesicles and then exosomes is largely unknown. Gurunathan et al. proposed that during exosome generation, EpCAM expressed on the plasma membrane probably translocates to exosomes through membrane invagination [109]. However, this hypothesis is challenged by how EpCAM returns to its normal state, because EpEX is located inside the endosome and EpICD outside the endosome when EpCAM undergoes membrane invagination. Recently, Leblanc et al. discovered that the pharmacological inhibition of the PDZ2 domain of syntenin with chemical inhibitors is able to severely reduce the sorting of EpCAM into exosomes, suggesting the involvement of syntenin in EpCAM translocation to exosomes [110]. However, no more evidence has been found to determine whether syntenin regulates EpCAM sorting into exosomes directly or indirectly. Additionally, it is worth noting that EpCAM has been proposed to possess a putative PDZ-binding site, though this is not experimentally confirmed [27]. It is possible that the inhibitor disrupts the interaction between the PDZ2 domain of syntenin and the LNA motif within the C-terminal of EpCAM.

In addition, exosomes are reported to be able to influence the expression of EpCAM on adjacent cells. Some hybrid or chimera cells were detected when human mesenchymal stem cells (MSCs) were cocultured with SK-OV-3 cells (ovarian cancer cells) or OVCAR-3 cells (ovarian adenocarcinoma cells). Meanwhile, nanotube structures, exosomes, and a significant increase in EpCAM expression were observed on MSCs after coculture [111]. Moreover, a recent study discovered that exosomes from liver stem cells (LSCs) can alleviate liver fibrosis in mice, while increasing the expression of EpCAM on LSCs [112].

### 4.3. Genetic and Epigenetic Regulation of EpCAM

Genetic regulation is a classical method of regulating the expression of genes, including *EPCAM*. Previous studies have reported that the deletion of the *EPCAM* gene is associated with CTE and Lynch syndromes through different mechanisms [64,113]. CTE syndrome is involved in the dysfunction of the intestine caused by EpCAM malfunction [114,115], whereas Lynch syndrome is linked to the promoter hypermethylation in downstream *MLH1* and *MHL2* genes induced by the deletion of 3’ end of EpCAM [113,116]. Recently, our work revealed that amplification of the *EPCAM* gene leads to its high expression in primary lung cancer [117]. Moreover, the data suggest that such amplification is present in approximately 41% of lung cancer patients. Cigarette smoking, the major cause of lung cancer, also significantly contributes to the amplification of the *EPCAM* gene and its strong expression [117].

Epigenetic regulation is another common mechanism to control the expression of EpCAM in various cancer tissues and cells (Table 1). The methylation status within the *EPCAM* promoter has been discussed in colon, ovarian, and breast cancer tissues and cells [118,119,120]. Van der Gun et al. discovered that EpCAM repression caused by DNA methyltransferases (DNMTs) is much more profound and persistent than that induced by siRNA interference in ovarian cancer cells [121]. Subsequent studies have indicated that EpCAM silencing is linked to its promoter methylation in oral squamous cell carcinoma [122] and metastatic lung cancer [117]. In addition, EpCAM is strongly upregulated in primary lung cancer but downregulated in metastatic lung cancer, which can be attributed to the shift of *EPCAM* promoter hypomethylation in primary lung cancer to *EPCAM* promoter hypermethylation in metastatic lung cancer [117]. In contrast, Shiah et al. found that DNMT1 is upregulated concurrently with EpCAM expression in oral squamous cell carcinoma, but no significant correlation between DNMT1 expression and *EPCAM* promoter methylation has been detected [122]. It seems that other DNMT family member(s) and/or the localization of DNMT1 in the *EPCAM* promoter contributes to the regulation of EpCAM expression. Interestingly, experiments independently conducted by Cui et al. and Tai et al. have demonstrated that the repression of EpCAM in metastatic lung cancer can be reversed by the DNMT inhibitor 5-aza-dC in a time- and dose-dependent manner [117,120].

In addition to promoter methylation, histone methylation and acetylation also play a significant role in epigenetic regulation. As mentioned before, EpCAM is expressed in undifferentiated hESCs but is rapidly silenced upon hESC differentiation. Lu et al. found that the silencing of EpCAM does not correlate with its promoter methylation but correlates with transcriptional repression mediated by histone H3K27 trimethylation, which is controlled by histone methyltransferase SUZ12 and histone demethylase JMJD3 [68]. A similar finding related to the dynamic regulation of H3K27m3 in *Epcam* loci is observed in mice [123]. The enzymes responsible for histone modification are also subject to regulation by other proteins. A recent study revealed that by inhibiting lysine demethylase 2A (KDM2A)-mediated demethylation of H3K26m2, ZHX2 downregulates the expression of stemness genes, including *EPCAM*, in hepatocellular carcinoma stem cells [125]. Histone methyltransferase G9a exhibits a negative association with EpCAM expression [126] because G9a is able to dimethylate histone H3K9, leading to the assembly of transcription repressor in the *EPCAM* promoter to ultimately suppress EpCAM expression in metastatic lung cancer [128,129]. Additionally, histone acetylation plays a critical role in the expression regulation of EpCAM. Upon treatment with histone deacetylase inhibitors (HDACis), remarkable induction of EpCAM expression is detected in metastatic lung cancer cells [117,120]. It is of importance to note that epigenetic regulation frequently synergizes with other modifications to regulate gene expression. For instance, the silencing of tumor suppressors DSC3 and MASPIN is caused by H3K9 dimethylation and subsequent promoter methylation [130]. Earlier studies have indicated that promoter methylation does not play a major role in regulating EpCAM expression in breast and colorectal cancers, implying multiple mechanisms for EpCAM expression regulation [118,119]. Accordingly, Tai et al. revealed the accumulation of heterochromatin protein 1 (HP1), H3K9 methyltransferase Suv39h1, HDAC1, DNMT1, and DNMT3b in the *EPCAM* promoter, along with the invasiveness of lung cancer cells [120]. Chen et al. also found that H3K9 dimethylation by G9a increases the recruitment of DNMT1 and HDAC1 to the *EPCAM* promoter [126]. Taken together, the downregulation of EpCAM in metastatic lung cancer results from a combination of DNA methylation, histone H3K9 methylation, and histone deacetylation.

In addition to both genetic regulation and epigenetic regulation by various methods, transcription factors play a crucial role in gene expression [131]. Van der Gun et al. identified various transcription factors involved in the regulation of EpCAM expression in ovarian cancer [124]. In other cancers, transcription factors, including LEF1 [52], Sp1 [132], NF-κB [133], STAT3 [134], and ETS1 [135], have been demonstrated to be indispensable for EpCAM expression. Overall, the expression of EpCAM is coordinated by transcription factors and genetic and epigenetic mechanisms in a context-specific manner.

### 4.4. The Clinical Application of EpCAM

In non-cancer diseases, the disturbance in EpCAM expression is also linked to function and phenotype. In CTE, for instance, the absence of or reduction in EpCAM severely affects intestinal epithelial cell-to-cell adhesion and consequently impairs intestinal absorption [64,136]. In addition, EpCAM reduction exacerbates the progression of inflammatory bowel disease (IBD), but its elevation is detrimental in cholestatic liver injury [136]. Moreover, EpCAM expression is found to be positively associated with the progression of nonalcoholic fatty liver disease (NAFLD). Elevated levels of EpCAM and CD133-positive exosomes indicate a transition from simple steatosis to steatohepatitis [137]. However, EpCAM targeting has not been applied to the treatment of non-cancer diseases.

As discussed before, the use of targeting EpCAM in CTC and exosome capture has been well addressed (Table 2). The clinical significance of CTC-based assays in aiding cancer diagnosis and prognosis has been well documented by Lin et al. [138]. In the context of clinical treatment, EpCAM is frequently used as a means of directing the precise delivery of drugs or small interfering RNA chimeras (AsiCs) to the lesion. For example, tucotuzumab celmoleukin, oportuzumab monatox, and citatuzumab bogatox have demonstrated promising clinical outcomes in phase I/II [139]. For therapeutic resistance, the mechanism by which EpCAM promotes tumor cell stemness may contribute to the development of resistance to conventional cancer therapies. Previous studies have demonstrated that EpCAM knockdown results in increased sensitivity to chemotherapy and radiotherapy in prostate cancer cells, which is interpreted by inactivation of PI3K/AKT/mTOR signaling [58]. Consistently, EpCAM has been shown to promote a more aggressive and drug-resistant phenotype through the activation of the AKT pathway in ovarian and nasopharyngeal cancers [59,140]. Similarly, EpCAM upregulation of AKT downstream targets promotes stemness and DNA loss repair in breast cancer cells, thereby enhancing resistance to radiotherapy and DNA-damaging chemotherapeutic agents (such as doxorubicin, cisplatin, or gemcitabine) [141]. However, the rapid formation of a large number of drug-resistant clones in AKT and mTOR inhibitor-treated xenograft demonstrates the complexity of EpCAM-mediated resistance mechanisms [142]. This is corroborated by the evidence that cisplatin resistance in tumor cells is induced by an EpCAM–claudin–tetraspanin complex [143] and an Nrf2–EpCAM axis [144]. Furthermore, the crosstalk of EpCAM with other pathways and cytokines is a crucial factor in the development of resistance [145]. For instance, RIP hydrolysis of EpCAM may induce resistance to cetuximab in EGFR-high head and neck squamous cell carcinomas [146,147].

The observation of EpCAM-mediated resistance to radiotherapy and chemotherapy suggests the potential of targeted EpCAM immunotherapy in cancer treatment. The efficacy and safety of early anti-EpCAM mAbs (e.g., adecatumumab and edrecolomab) have not been as desired clinically, leading to the development of bispecific antibodies (BsAbs) [7]. Among these, catumaxomab is the world’s first trifunctional bispecific antibody (Triomab) approved for the treatment of malignant ascites. There were also phase II clinical trials completed in EpCAM-positive solid tumors, such as gastric and ovarian cancers, with favorable outcomes and acceptable side effects [157]. In contrast, solitomab (MT110), also targeting EpCAM and CD3, is a type of bispecific T-cell engaging antibody (BiTE) that is more permeable and has a shorter half-life compared to Triomab. MT110 is capable of stimulating T-cell activation to kill uterine and ovarian cancer cells [158], as well as pancreatic cancer stem cells [159]. The phase I study (NCT00635596) was completed, and a phase II trial is ongoing. In cellular immunotherapy, bispecific CAR-T cells targeting EpCAM and intercellular adhesion molecule 1 (ICAM-1) have demonstrated remarkable efficacy [160], while EpCAM-CAR-NK92 cells exhibit a synergistic therapeutic effect with regorafenib in colon cancer [161].

## 5. Conclusions

EpCAM has emerged as a promising target in cancer diagnosis and treatment, including as a cell surface marker to isolate CTCs and exosomes and to design CAR-T cell therapy [162,163,164]. Although great progress has been made in the past few decades, the context-specific expression profile of EpCAM and its regulation urgent need to be urgently determined for the application of EpCAM-based techniques in both basic and translational cancer research. Given that various mechanisms are involved in the regulation of EpCAM expression, including chromatin (DNA and histone) modification and transcription factor, it seems plausible to use them to broaden the application of current EpCAM-based products. For example, the epigenetic restoration of EpCAM expression in metastatic lung cancers by HDACi and/or DNMTi probably enhances the yield of CTC isolation and the specific killing of cancer cells by anti-EpCAM CAR-T therapy. Further studies are also required to determine the structure of EpCAM to develop novel antibodies, contributing to specific and sensitive strategies for the diagnosis and treatment of human diseases. 

## Figures and Tables

**Table 1:** Regulation of EpCAM expression in various tissues and cells.

Tissue/Cell Type	Regulation	Expression	Reference
Colorectal cancer cells	DNA methylation, H3K27 trimethylation	↓	Yu et al. [118], Liao et al. [123]
Human breast cancer cells	DNA methylation	↓	Spizzo et al. [119]
Ovarian cancer cells	DNA methylation, histone methylation	↓	Van der Gun et al. [121,124]
Oral squamous cell carcinoma	?	↑	Shiah et al. [122]
Human embryonic stem cells	H3K27 trimethylation	↓	Lu et al. [68]
Liver cancer stem cells	H3K26 dimethylation	↓	Lin et al. [125]
Primary lung cancer	Gene amplification	↑	Cui et al. [117]
Metastatic lung cancer	DNA methylation, histone deacetylation, H3K9 dimethylation	↓	Tai et al. [120], Chen et al. [126], Lin et al. [127]

↓ = downregulation, ↑ = upregulation, ? = unknown.

**Table 2:** Selected clinical applications of EpCAM in recent three years (from 2022 to 2024).

Tumor Type	Diagnostic Methods	Prognostic Value
Epithelial cancers	EpCAM- and MUC1-positive small cell vesicles [148]	Not updated
High-grade serous ovarian cancer	EpCAM, CD24, VCAN, HE4, and TNC as markers of exocytotic vesicles (89% sensitivity and 93% specificity) [149]	EpCAM mRNA expression is correlated with longer overall survival (HR = 0.89, 95%, 0.80–0.99; *p* = 0.039) [150].
Epithelial ovarian cancer	An optimized detection model for CTCs expressing EpCAM, MUC1, and WT1 (79.4% sensitivity and 92.2% specificity) [151]	EpCAM^+^ CTCs has predictive value for chemotherapy resistance (*p* < 0.05) [151].
Osteosarcoma	Integrated microfluidic-SERS for exosomes ex-pressing CD63, VIM, and EpCAM (sensitivity, specificity, and accuracy of 100%, 90% and 95%, respectively) [152]	Not updated.
Hepatocellular carcinoma	Not updated	Combining EpCAM^+^ CTCs and AFP identifies patients with poor outcomes after surgical resection [153].
Prostate cancer	One-step thermophoretic AND gate operation on extracellular vesicles expressing EpCAM and PMSA (accuracy of 91%) [154]	EpCAM expression is negatively correlated with prostate cancer prognosis [155].
Non-small-cell lung cancer	Ratiometric electrochemical OR gate assay for exosomes expressing CEA and EpCAM (93.3% sensitivity) [156]	CEA and EpCAM expression is highly predictive for cancer recurrence (AUC of 1.000) [156].

## Data Availability

Any data related to the work are available upon request.
